# Early vs Late Fixation of Extremity Fractures Among Adults With Traumatic Brain Injury

**DOI:** 10.1001/jamanetworkopen.2024.1556

**Published:** 2024-03-08

**Authors:** Jiang Zheng, Yufang Ouyang, Ke Zhang, Zhixing Wang, Alexander Younsi, Obada Alhalabi, Hong Fu

**Affiliations:** 1 Department of Anesthesiology, Chongqing Emergency Medical Center, Chongqing University Central Hospital, School of Medicine, Chongqing University, Chongqing, China; 2 Department of Neurosurgery, University Hospital Heidelberg, Heidelberg, Germany

## Abstract

**Importance:**

The optimal timing for fixation of extremity fractures after traumatic brain injury (TBI) remains controversial.

**Objective:**

To investigate whether patients who underwent extremity fixation within 24 hours of TBI experienced worse outcomes than those who had the procedure 24 hours or more after TBI.

**Design, Setting, and Participants:**

This cohort study used data from the Collaborative European NeuroTrauma Effectiveness Research in Traumatic Brain Injury (CENTER-TBI) study. Patients 16 years or older with TBI who underwent internal extremity fixation met inclusion criteria. To compare outcomes, patients who underwent the procedure within 24 hours were propensity score matched with those who underwent it 24 hours or later. Patients were treated from December 9, 2014, to December 17, 2017. Data analysis was conducted between August 1, 2022, and December 25, 2023.

**Main Outcomes and Measures:**

The primary outcome was an unfavorable functional status at 6 months (Glasgow Outcome Scale–Extended [GOSE] score ≤4).

**Results:**

A total of 253 patients were included in this study. The median age was 41 (IQR, 27-57) years, and 184 patients (72.7%) were male. The median Injury Severity Score (ISS) was 41 (IQR, 27-49). Approximately half of the patients (122 [48.2%]) had a mild TBI while 120 (47.4%) had moderate to severe TBI. Seventy-four patients (29.2%) underwent an internal extremity fixation within 24 hours, while 179 (70.8%) had the procedure 24 hours or later. At 6 months, 86 patients (34.0%) had an unfavorable functional outcome. After propensity score matching, there were no statistically significant differences in unfavorable functional outcomes at 6 months (odds ratio [OR], 1.12 [95% CI, 0.51-1.99]; *P* = .77) in patients with TBI of any severity. Similar results were observed in patients with mild TBI (OR, 0.71 [95% CI, 0.22-2.29]; *P* = .56) and moderate to severe TBI (OR, 1.08 [95% CI, 0.32-3.70]; *P* = .90).

**Conclusions and Relevance:**

The outcomes of extremity fracture fixation performed within 24 hours after TBI appear not to be worse than those of procedures performed 24 hours or later. This finding suggests that early fixation after TBI could be considered in patients with mild head injuries.

## Key Points

**Question:**

Are functional outcomes different for patients with traumatic brain injury who undergo early (within 24 hours of injury) vs late extremity fixation?

**Findings:**

In this cohort study including 253 patients, there were no statistically significant differences in unfavorable functional outcomes at 6 months between the early and late extremity fixation groups.

**Meaning:**

These findings suggest that early extremity fixation could be considered an option for patients with traumatic brain injury.

## Teaser

This cohort study assesses the 6-month functional outcomes of patients with traumatic brain injury who undergo early (within 24 hours of injury) vs late extremity fixation.

## Introduction

Traumatic brain injury (TBI) is a major cause of death and disability worldwide.^1^ Severe extracranial injury to the extremities, chest, and abdomen is found in about 30% to 50% of TBI cases. Indeed, head injuries combined with extremity fractures simultaneously are common in high-energy traumas.^2,3,4^ While early surgical intervention for fracture reposition might be indicated in many cases, the ideal timing for the fixation of extremity fractures in patients with TBI remains controversial, with advocates of early fixation citing reduced mortality, decreased complications, and shorter intensive care unit (ICU) stays.^5,6,7^ However, concerns have been expressed that early extremity fixation (EEF) could increase the risk of secondary brain damage because of intraoperative adverse events (ie, blood loss, hypoxia, and hypotension) or coagulopathy and the inflammatory response.^8^ Although a previous meta-analysis of retrospective studies^9^ found no significant differences in mortality, medical complications, or adverse neurological events when comparing fixation within 24 hours of TBI and fixation occurring 24 hours or later, that analysis only included retrospective studies conducted decades ago. Furthermore, previous studies did not include assessment of longer-term functional outcomes by, for example, the Glasgow Outcome Scale–Extended (GOSE) score. As such, evidence to guide clinicians on the timing of extremity fixation in patients with TBI is still scarce.

Hence, our current analysis assesses different functional outcomes of patients with TBI who undergo EEF (within 24 hours of injury) vs late extremity fixation (LEF; 24 hours after injury or later). We hypothesize that functional outcomes of patients who undergo EEF are not worse than those of patients who undergo LEF.

## Methods

### Study Population and Design

The population of this cohort study stems from the multicenter, longitudinal, prospective CENTER-TBI (Collaborative European NeuroTrauma Effectiveness Research in Traumatic Brain Injury) study, which included patients with TBI of all severities and a clinical indication for brain computed tomography (CT) who presented to 65 centers in Europe and Israel within 24 hours after the injury from December 9, 2014, to December 17, 2017.^10^ The CENTER-TBI study was approved by the Medical Ethics Committees of all participating centers, and written informed consent was obtained from all patients prior to enrollment, in accordance with local regulations. For our current analysis, we included all patients from the CENTER-TBI core study dataset who were 16 years or older at the time of injury and underwent internal extremity fixation (IEF) after TBI. Patients who did not undergo surgery and only received external extremity fixation were excluded. The patients who underwent IEF were divided into the EEF group, who received the treatment within 24 hours after TBI, and the LEF group, who received treatment 24 hours or later after TBI. The study followed the Strengthening the Reporting of Observational Studies in Epidemiology (STROBE) reporting guideline.

### Data Collection

The CENTER-TBI study collected comprehensive information on demographic characteristics, injury characteristics, clinical profiles, laboratory characteristics, monitoring, treatment intensity levels, and outcomes. Additional details can be found in previous studies.^10,11^

### Outcomes

The primary outcome was functional status at 6 months, which was assessed using the GOSE. The GOSE is measured on a scale from 1 to 8; a score of 1 represents death, while a score of 8 indicates upper-good recovery.^12^ The GOSE scores were measured by trained personnel.^10^ Patients with a GOSE score of 4 or lower were classified as having an unfavorable outcome. The secondary outcomes included in-hospital, 30-day, and 6-month mortality rates; length of stay (LOS); and complications such as respiratory complications, cardiovascular complications, raised intracranial pressure (ICP), urinary tract infection (UTI), delayed hematoma, metabolic complications, seizures, and deep vein thrombosis.

### Statistical Analysis

Continuous variables are reported as mean (SD) or median (IQR); categorical variables are reported as counts (percentages). The differences between groups were assessed using the unpaired *t* test or Mann-Whitney test for continuous variables, depending on the data distribution. For categorical variables, a χ^2^ test or Fisher exact test was used.

Multiple imputation was used to account for missing data, and 5 imputed datasets were obtained. The missingness of the data was assumed to be random. All analyses were conducted on each dataset separately, and the results were pooled according to Rubin rules. Propensity score matching (PSM) was performed to balance baseline covariates between the EEF and LEF groups controlled by these variables (age; sex; total Injury Severity Score [ISS], which ranges from 1 to 75 [with higher scores indicating greater severity]; lower extremity Abbreviated Injury Scale [AIS] score, which ranges from 0 to 5 [with higher scores indicating greater severity]; Glasgow Coma Scale [GCS] score, which ranges from 3 to 15 [with lower scores indicating more severe coma]; pupillary reactivity; preinjury American Society of Anesthesiologists physical status classification; cranial surgery; and abnormal CT finding), with a 1:1 match and 0.2 caliper. Subgroup analysis was performed by dividing patients into 2 groups based on the severity of head injury: moderate to severe TBI (GCS score ≤12) and mild TBI (GCS score >12). Propensity score matching was also performed separately in each subgroup. Furthermore, multivariable logistic regression was performed as a complementary analysis. The EEF or LEF was tested as a factor associated with outcome, along with the variables with *P* < .05 in the univariable logistic regression or clinically relevant potential risk factors for unfavorable outcomes, to determine the association of timing with unfavorable outcome.^13^

All statistical analyses were performed using SPSS, version 26.0 (IBM Corporation), and R, version 3.5.1 (R Project for Statistical Computing). A 2-sided significance level of *P* = .05 was used for hypothesis testing, with no adjustments for multiple comparisons. This analysis was conducted from August 1, 2022, to December 25, 2023.

## Results

### Patient Characteristics

Of the 4509 participants in the CENTER-TBI core study, 305 who were 16 years or older underwent extremity fixation, and 50 with external fixation only and 2 without surgery time were excluded. The remaining 253 patients underwent IEF and were included in our study (Figure). Table 1 displays the characteristics of 253 patients with TBI by timing of extremity fixation in the unmatched cohort. Of the included patients, 74 (29.2%) underwent extremity fixation within 24 hours of TBI. Among these, 30 patients (40.5%) had moderate to severe head injury, 39 (52.7%) had mild injury, and 5 (6.8%) had missing head injury information. On the other hand, 179 patients (70.8%) underwent extremity fixation 24 hours or more after the injury. Of this group, 90 patients (50.3%) had moderate to severe head injury, 83 (46.4%) had mild injury, and 6 (3.4%) had missing head injury information. More details can be found in eTables 1 and 3 in Supplement 1. Most of the 253 patients were male (184 [72.7%] compared with 69 [27.3%] female). Racial and ethnic data were not collected because no strong evidence suggests that these characteristics have an impact on the functional outcomes of patients after TBI. More than one-half of patients (145 [57.3%]) were admitted to the ICU, and almost half (122 [48.2%]) had a mild TBI (GCS, 13-15) or a moderate to severe TBI (120 [47.4%]). The median ISS was 41 (IQR, 27-49), 229 patients (90.5%) had a severe injury (ISS >16), 113 (44.7%) had a severe upper extremity injury, and 134 (53.0%) had a severe lower extremity injury (an ISS ≥3 in the upper and lower extremity). Most patients (209 [82.6%]) had normal reactivity in both pupils, and 159 (62.8%) had a preinjury healthy status. About one-fifth of patients (49 [19.4%]) underwent cranial surgery, and 183 (72.3%) had an abnormal intracranial CT finding.

**Figure.**
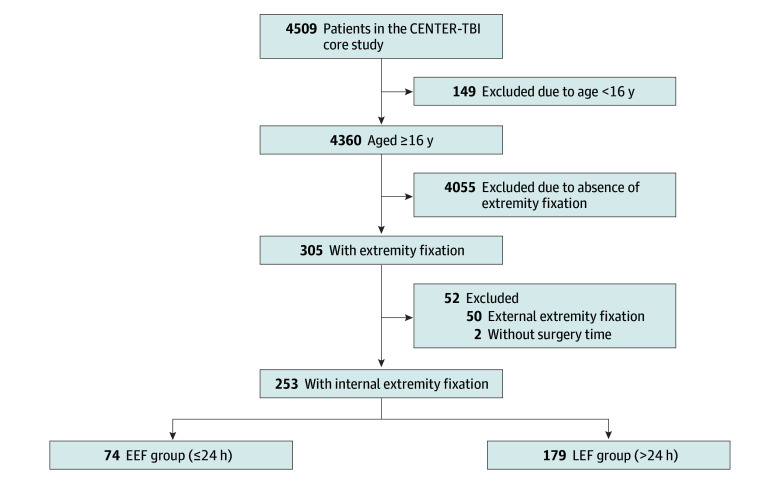
Flow Diagram of Study Population CENTER-TBI indicates Collaborative European NeuroTrauma Effectiveness Research in Traumatic Brain Injury; EEF, early extremity fixation; and LEF, late extremity fixation.

**Table 1.** Characteristics of Patients With Traumatic Brain Injuries by Timing of Extremity Fixation in the Unmatched Cohort^a^

Characteristic	Patient group			*P* value
	All (N = 253)	EEF (n = 74)	LEF (n = 179)	
Age, median (IQR), y	41 (27-57)	40 (25-56)	40.5 (27.0-58.0)	.32
Sex				
Female	69 (27.3)	27 (36.5)	42 (23.5)	.03
Male	184 (72.7)	47 (63.5)	137 (76.5)	
Clinical care pathway				
ED	1 (0.4)	1 (1.4)	0	.18
Admission	44 (17.4)	10 (13.5)	34 (19.0)	
ICU	208 (82.2)	63 (85.1)	145 (81.0)	
ISS, median (IQR)^b^	41 (27-49)	41.0 (26.3-48.0)	41.0 (27.0-50.0)	.80
ISS^b^				
≤16	20 (7.9)	7 (9.5)	13 (7.3)	.56
>16	229 (90.5)	66 (89.2)	163 (91.1)	
Upper extremity AIS score^c^				
<3	140 (55.3)	40 (54.1)	100 (55.9)	.79
≥3	113 (44.7)	34 (45.9)	79 (44.1)	
Lower extremity AIS score^c^				
<3	119 (47.0)	30 (40.5)	89 (49.7)	.18
≥3	134 (53.0)	44 (59.5)	90 (50.3)	
GCS score				
Mild (13-15)	122 (48.2)	39 (52.7)	83 (46.4)	.23
Moderate (9-12)	30 (11.9)	7 (9.5)	23 (12.8)	
Severe (3-8)	90 (35.6)	23 (31.1)	67 (37.4)	
GCS motor score, median (IQR)^d^	5 (1-6)	6 (1.5-6)	5 (1-6)	.22
Pupillary reactivity				
Both reactive	209 (82.6)	64 (86.5)	145 (81.0)	.92
1 Reactive	12 (4.7)	3 (4.1)	9 (5.0)	
Both nonreactive	15 (5.9)	5 (6.8)	10 (5.6)	
Preinjury ASA PS				
Healthy	159 (62.8)	49 (66.2)	110 (61.5)	.42
Mild systemic disease	78 (30.8)	20 (27.0)	58 (32.4)	
Severe systemic disease	8 (3.2)	1 (1.4)	7 (3.9)	
Unknown	8 (3.2)	4 (5.4)	4 (2.2)	
Cranial surgery				
Yes	49 (19.4)	15 (20.3)	34 (19.0)	.82
No	204 (80.6)	59 (79.7)	145 (81.0)	
Abnormal CT finding				
Yes	183 (72.3)	46 (62.2)	137 (76.5)	.01
No	66 (26.1)	28 (37.8)	38 (21.2)	
Uninterpretable	4 (1.6)	0	4 (2.2)	

Abbreviations: AIS, Abbreviated Injury Scale; ASA PS, American Society of Anesthesiologists physical status; CT, computed tomography; ED, emergency department; EEF, early extremity fixation; ICU, intensive care unit; ISS, Injury Severity Score; GCS, Glasgow Coma Scale; LEF, late extremity fixation.

^a^ Unless indicated otherwise, data are presented as No. (%) of patients. Owing to missing data, percentages may not total 100.

^b^ Scores range from 1 to 75, with higher scores indicating greater severity.

^c^ Scores range from 0 to 5, with higher scores indicating greater severity.

^d^ Scores range from 1 to 6, with higher scores indicating better motor function.

Before PSM, a significantly higher proportion of female patients underwent EEF compared with LEF (27 of 74 [36.5%] vs 42 of 179 [23.5%]; *P* = .03). Moreover, patients with abnormal findings on CT scans were more likely to undergo LEF (137 [76.5%] vs 38 [21.2%]; *P* = .01), especially in the mild TBI group (54 of 83 [65.1%] vs 17 of 39 [43.6%]; *P* = .03) (Table 1 and eTable 3 in Supplement 1). The balance of covariates before and after matching can be assessed using standardized mean differences less than 0.1. After PSM, 69 patient pairs were formed in all severity levels of TBI, 29 in the moderate to severe TBI subgroup, and 35 in the mild TBI subgroup, with successfully balanced baseline characteristics (Table 2 and eTables 2 and 4 and eFigures 1 and 2 in Supplement 1).

**Table 2.** Characteristics of Patients With All Severity Traumatic Brain Injury by Timing of Extremity Fixation in the Propensity Score–Matched Cohort^a^

Characteristic	Patient group (N = 138)		*P* value	SMD	
	EEF (n = 69)	LEF (n = 69)		Before matching	After matching
Age, median (IQR), y	40 (25-55)	37 (26-55)	.69	0.12	0.09
Sex					
Female	23 (33.3)	23 (33.3)	.94	−0.27	−0.01
Male	46 (66.7)	46 (66.7)			
ISS, median (IQR), y^b^	41 (27-49)	38 (27-47)	.66	0.03	0.08
Lower extremity AIS score^c^			.95	0.19	0.01
<3	29 (42.0)	28 (40.6)	.92		
≥3	40 (58.0)	41 (59.4)			
GCS score					
Mild (13-15)	37 (53.6)	36 (52.2)	.80	0.17	0.05
Moderate to severe (3-12)	32 (46.4)	33 (47.8)			
Pupillary reactivity					
Both reactive	61 (88.4)	60 (87.0)	.96	0.04	0.08
1 Reactive	3 (4.3)	4 (5.8)		−0.08	−0.09
Both nonreactive	5 (7.2)	5 (7.2)		0.04	0.01
ASA PS					
Healthy	47 (68.1)	48 (69.6)	.90	0.14	0.02
Mild to severe systemic disease	22 (31.9)	21 (30.4)			
Cranial surgery					
Yes	55 (79.7)	54 (78.3)	.89	0.03	0.01
No	14 (20.3)	15 (21.7)			
Abnormal CT finding					
Yes	46 (66.7)	47 (68.1)	.77	0.29	0.03
No	23 (33.3)	22 (31.9)			

Abbreviations: AIS, Abbreviated Injury Scale; ASA PS, preinjury American Society of Anesthesiologists Physical Status; CT, computed tomography; EEF, early extremity fixation; GCS, Glasgow Coma Scale; ISS, Injury Severity Score; LEF, late extremity fixation; SMD, standardized mean difference.

^a^ Unless indicated otherwise, data are presented as No. (%) of patients.

^b^ Scores range from 1 to 75, with higher scores indicating greater severity.

^c^ Scores range from 0 to 5, with higher scores indicating greater severity.

### Outcomes

Of the 253 patients with TBI and IEF in this study, 86 (34.0%) had an unfavorable outcome at 6 months. Ten patients (4.0%) died in the hospital, 8 (3.2%) died within 30 days, and 15 (5.9%) died within 6 months after injury. The most common complications during the hospital stay were respiratory complications (54 [21.3%]), followed by raised ICP (34 [13.4%]), UTI (25 [9.9%]), and cardiac events (19 [7.5%]) (Table 3). The patients with moderate to severe head injury had a much higher rate of adverse events than patients with mild head injury (eg, unfavorable functional outcomes at 6 months, 48 [40.0%] vs 30 [24.6%]; respiratory complications, 35 [29.2%] vs 14 [11.5%]) (Table 3 and Table 4).

**Table 3.** Secondary Outcomes in Patients With TBI

Secondary outcome	TBI subgroup^a^		
	Moderate to severe (n = 120)	Mild (n = 122)	All (N = 253)
Mortality			
In-hospital	7 (5.8)	2 (1.6)	10 (4.0)
30 d	6 (5.0)	1 (0.8)	8 (3.2)
6 mo	10 (8.3)	4 (3.3)	15 (5.9)
Complications			
Respiratory	35 (29.2)	14 (11.5)	54 (21.3)
Cardiac	12 (10.0)	6 (4.9)	19 (7.5)
Raised ICP	26 (21.7)	7 (5.7)	34 (13.4)
UTI	18 (15.0)	6 (4.9)	25 (9.9)
Metabolic	10 (8.3)	3 (2.5)	15 (5.9)
Delayed hematoma	8 (6.7)	4 (3.3)	12 (4.7)
Seizures	6 (5.0)	4 (3.3)	10 (4.0)
DVT	5 (4.2)	3 (2.5)	9 (3.6)
LOS, median (IQR), d	27.0 (15.6-48.8)	15.9 (8.5-26.8)	21.1 (13.0-35.9)

Abbreviations: DVT, deep vein thrombosis; ICP, intracranial pressure; LOS, length of stay; TBI, traumatic brain injury; UTI, urinary tract infection.

^a^ Unless otherwise indicated, data are presented as No. (%) of patients. Patients without Glasgow Coma Scale score (n = 11) were not included.

**Table 4.** Unfavorable Functional Outcomes at 6 Months in All Patients and in Patients With Moderate to Severe and Mild TBI

TBI severity	Unfavorable functional outcome, No./total (%)			OR (95% CI)	*P* value
	Unmatched cohort^a^	Matched cohort			
		EEF	LEF		
All	86/253 (34.0)	28/69 (40.6)	25/69 (36.2)	1.12 (0.51-1.99)	.77
Moderate to severe	48/120 (40.0)	14/29 (48.3)	13/29 (44.8)	1.08 (0.32-3.70)	.90
Mild	30/122 (24.6)	10/35 (28.6)	12/35 (34.3)	0.71 (0.22-2.29)	.56

Abbreviations: EEF, early extremity fixation; LEF, late extremity fixation; OR, odds ratio; TBI, traumatic brain injury.

^a^ Patients without Glasgow Coma Scale scores (n = 11) were not included.

After PSM in all patients with TBI, we did not find significant differences between the EEF and LEF groups in terms of an unfavorable functional outcome (odds ratio [OR], 1.12 [95% CI, 0.51-1.99]; *P* = .77). Similar results were observed in the subgroup analysis including patients with moderate to severe TBI (OR, 1.08 [95% CI, 0.32-3.70]; *P* = .90) or mild TBI (OR, 0.71 [95% CI, 0.22-2.29]; *P* = .56), with no significant differences in unfavorable functional outcomes between the 2 groups (Table 4).

There were no significant differences between the EEF and LEF groups in secondary outcomes (eTable 5 in Supplement 1). The analysis showed no significant differences in 30-day mortality (3 [4.3%] vs 0; *P* = .25), in-hospital mortality (3 [4.3%] vs 0; *P* = .25), 6-month mortality (3 [4.3%] vs 3 [4.3%]; *P* = .98), respiratory complications (11 [15.9%] vs 17 [24.6%]; *P* = .28), cardiac complications (2 [2.9%] vs 6 [8.7%]; *P* = .18), raised ICP (10 [14.5%] vs 9 [13.0%]; *P* = .99), UTI (4 [5.8%] vs 6 [8.7%]; *P* = .52), delayed hematoma (5 [7.2%] vs 2 [2.9%]; *P* = .41), metabolic complications (2 [2.9%] vs 6 [8.7%]; *P* = .24), seizures (2 [2.9%] vs 4 [5.8%]; *P* = .57), and deep vein thrombosis (3 [4.3%] vs 4 [5.8%]; *P* = .84). Length of stay was also comparable between the groups (median, 20.0 [IQR, 12.3-35.8] vs 24.6 [IQR, 13.6-41.6] days; *P* = .18). Similarly, in the subgroup analysis of patients with mild and moderate to severe TBI, no statistically significant difference was seen in the aforementioned complications between the EEF group and LEF group (eTable 5 in Supplement 1). In the complementary analysis with multivariable logistic regression analysis adjusted for age, sex, ISS, lower extremity AIS score, pupil reactivity, GCS score, GCS motor score, American Society of Anesthesiologists physical status, cranial surgery, and abnormal CT finding, EEF vs LEF was not a significant risk factor for an unfavorable functional outcome at the 6 months (OR, 1.14 [95% CI, 0.58-2.25]; *P* = .70). In contrast, severe lower extremity injury (OR, 1.49 [95% CI, 1.07-2.06]; *P* = .001) and nonreactivity in both pupils (OR, 18.75 [95% CI, 2.06-170.85]; *P* = .01) were independent risk factors for an unfavorable functional outcome (eTable 6 in Supplement 1).

## Discussion

In this PSM cohort study, we compared patient outcomes between an EEF group with surgery within 24 hours after TBI and an LEF group with surgery 24 hours or later after the injury. Our findings suggest that EEF was not associated with a 6-month unfavorable functional outcome, mortality, complications, or longer LOS. These results align with those of a meta-analysis^9^ that reported no significant association of fixation performed within 24 hours after injury with mortality, pneumonia, acute respiratory distress syndrome, or adverse neurological events. Unfortunately, this meta-analysis did not include longer-term functional outcomes, which are an important prognostic variable of patients after TBI highly valued by clinicians.

One major concern with EEF is the potential risk for secondary insults in the injured brain, which have been linked to poor neurological outcomes and mortality in patients with TBI.^14^ In the early stages of trauma, the damaged brain is particularly susceptible to ischemia and hypotension, and surgical intervention and anesthesia can increase the risk of such secondary insults owing to blood loss and intraoperative hypotension.^15,16^ Additionally, internal fixation may lead to increased ICP and decreased cerebral perfusion pressure, which frequently occurs in patients after severe TBI.^17^ In our study, 34 patients (13.4%) had an increased ICP during their hospital stay, but we found no differences between the EEF and LEF groups. Our results thus suggest that performing an extremity fixation in the early stage after TBI may not necessarily increase the risk of increased ICP. This might be related to advancements in anesthesiology and surgical techniques that could provide more opportunities for EEF after TBI. Propofol can support cerebral perfusion pressure and mean arterial pressure while reducing ICP, cerebral blood flow, cerebral metabolism, and edema. Hyperventilation can also decrease ICP by inducing hypocapnia.^18,19^

Previous studies^20,21^ have found that (1) early femur fracture fixation was associated with better outcomes, (2) patients who underwent LEF had higher rates of mortality and morbidity and longer ICU stays; and (3) the same results occurred in patients who also had head injuries. However, in the present study, we did not find significant differences in outcomes between the LEF and EEF groups. Some patients in our cohort may have been treated with damage control orthopedics, which is an option in patients with multiple traumas and provides provisional stability, usually through external fixation.^22,23^ Data on this strategy are not available in the CENTER-TBI study database but may have contributed to better outcomes in the LEF group. However, delayed treatment is often a result of worse physiologic status, which may be characterized by a lower GCS, a higher ISS or higher AIS score, and multiple preinjury medical comorbidities. These factors are associated with worse outcomes and may have influenced the timing of extremity fixation.^21^ Additionally, for patients with moderate to severe TBI, the severity of head injury is a risk factor associated with mortality and poor outcomes.^24^ In our cohort, 8 patients died within 30 days, most of whom had a GCS score of 3, indicating a serious head injury. While head injury severity, rather than delayed surgery, may contribute to worse outcomes in general, this was well balanced between the 2 groups in our PSM study and should not have affected our analysis.

Approximately one-third of patients undergoing IEF after TBI in our analysis had an unfavorable outcome at the 6-month follow-up, which is higher than the incidence of unfavorable outcomes in the entire CENTER-TBI study cohort (24%).^25^ It is noteworthy that approximately half of the patients we assessed had a severe extremity injury with a lower extremity AIS score of 3 or higher (53.0%), upper extremity AIS score of 3 or higher (44.7%), and moderate to severe TBI (47.4%). Additionally, 90.5% of the patients in our cohort had severe injuries (ISS >16) beyond isolated TBI, which might have contributed to their longer-term functional status. Nevertheless, in comparison with the entire CENTER-TBI study cohort, our population of patients undergoing IEF had a lower GCS score as well, which has been associated with worse outcomes.^26^ This could be a surrogate for the severeness of the injury rather than the surgery itself being responsible for a worse outcome. There was no statistically significant difference in unfavorable outcomes between the LEF and EEF groups after PSM. Furthermore, in a supplementary multivariable logistics regression analysis, after adjusting for baseline and injury characteristics, we found that timing of extremity fixation was not a risk factor for an unfavorable functional outcome, while AIS of the lower extremity was. The GOSE score can be confounded by nonneurological injury; the nature of a severe injury to the lower limbs could constitute a reduced ambulatory status that reduces a patient’s GOSE score but may be due to orthopedic mechanical rather than neurological reasons. However, it is difficult to disentangle the effects of systemic injuries from those of brain injuries in clinical practice.^27^ Roberts et al^28^ found a correlation between extracranial surgery and anesthesia with adverse functional outcomes and impaired executive function following TBI. Nevertheless, it is vital to identify patients with lower extremity injuries and ensure that proper treatment options are available, regardless of the time of injury.

### Strengths and Limitations

Our study has 2 main strengths. First, although only 253 patients were included, to our knowledge they represent the largest sample of multicentric, longitudinal, prospectively collected data on external fixation after TBI to date. Previous studies were retrospective and had smaller sample sizes. Second, we used PSM to adjust for confounders, which allowed for better comparison between the EEF and LEF groups.

This study also has some limitations. The CENTER-TBI core study mainly focused on TBI and lacks detailed information on extracranial operations and associated intraoperative events, such as intraoperative hypotension or blood loss, which may have implications for the prognosis; therefore, our findings should be interpreted with caution. Additionally, our PSM method, although comprehensive, may not have accounted for unobserved variables. For instance, factors such as the experience and opinions of the surgeon can play a role in the decision to perform surgery, yet they may not be captured in the clinical data.^29^ Other factors such as surgical urgency,^30^ insults introduced intraoperatively or during transportation, and other factors that may affect the results were not considered in our analysis.^28^ Patients with severe TBI (GCS score <8) are more likely to experience secondary insults (eg, hypotension, intraoperative bleeding, hypoxia, hyperventilation, and increase in ICP) due to lying flat on the operating table; these patients are least likely to benefit from early ambulation and shorter bed rest. For patients with moderate to severe TBI, early fixation may increase the risk of secondary insult, suggesting that there is no need to expedite extremity fracture fixation. However, for patients with mild TBI, who are less likely to experience adverse events, EEF or LEF may not have significant differences in terms of outcomes; in fact, EEF may provide more benefits for these patients. Regardless, a robust evidence base for determining the best timing of extremity fracture surgery after TBI is lacking, and it is difficult to design randomized clinical trials for this circumstance.

## Conclusions

In this PSM cohort analysis of patients with TBI undergoing IEF, early surgery within 24 hours of the injury did not result in worse outcomes compared with later surgery. Thus, in selected patients with mild head injuries, EEF could be considered. Future research should prospectively collect more information on surgery details and intraoperative events and include larger sample sizes to derive more reliable conclusions.

## Notes

Supplement 1. **eTable 1.** Characteristics of 120 Patients With Moderate to Severe TBI by Timing of Extremity Fixation, Unmatched Cohort **eTable 2.** Characteristics of 58 Patients With Moderate to Severe TBI by Timing of Extremity Fixation, Matched Cohort **eTable 3.** Characteristics of 122 Patients With Mild TBI by Timing of Extremity Fixation, Unmatched Cohort **eTable 4.** Characteristics of 70 Patients With Mild TBI by Timing of Extremity Fixation, Matched Cohort **eTable 5.** Secondary Outcomes of Patients With TBI by Timing of Extremity Fixation **eTable 6.** Univariable and Multivariable Analysis for an Unfavorable Functional Outcome After 6 Months **eFigure 1.** Covariate Balance Following Propensity Score Matching **eFigure 2.** Distribution of Propensity Scores

Supplement 2.
Nonauthor Contributors

Supplement 3.
Data Sharing Statement
