# Supplementary material for: Early vs Late Fixation of Extremity Fractures Among Adults With Traumatic Brain Injury
Source: JAMA Netw Open. 2024 Mar 8;7(3):e241556. doi: 10.1001/jamanetworkopen.2024.1556 (PMC10924246; doi:10.1001/jamanetworkopen.2024.1556)
Supplement: Supplement 1. — eTable 1. Characteristics of 120 Patients With Moderate to Severe TBI by Timing of Extremity Fixation, Unmatched Cohort eTable 2. Characteristics of 58 Patients With Moderate to Severe TBI by Timing of Extremity Fixation, Matched Cohort eTable 3. Characteristics of 122 Patients With Mild TBI by Timing of Extremity Fixation, Unmatched Cohort eTable 4. Characteristics of 70 Patients With Mild TBI by Timing of Extremity Fixation, Matched Cohort eTable 5. Secondary Outcomes of Patients With TBI by Timing of Extremity Fixation eTable 6. Univariable and Multivariable Analysis for an Unfavorable Functional Outcome After 6 Months eFigure 1. Covariate Balance Following Propensity Score Matching eFigure 2. Distribution of Propensity Scores [file jamanetwopen-e241556-s001.pdf]

Supplementary Online Content

Zheng J, Ouyang Y, Zhang K, et al; Collaborative European NeuroTrauma Effectiveness Research in Traumatic Brain Injury (CENTER-TBI) Investigators. Early vs late fixation of extremity fractures among adults with traumatic brain injury. *JAMA Netw Open*. 2024;7(3):e241556. doi:10.1001/jamanetworkopen.2024.1556

eTable 1. Characteristics of 120 Patients With Moderate to Severe TBI by Timing of Extremity Fixation, Unmatched Cohort
eTable 2. Characteristics of 58 Patients With Moderate to Severe TBI by Timing of Extremity Fixation, Matched Cohort
eTable 3. Characteristics of 122 Patients With Mild TBI by Timing of Extremity Fixation, Unmatched Cohort
eTable 4. Characteristics of 70 Patients With Mild TBI by Timing of Extremity Fixation, Matched Cohort
eTable 5. Secondary Outcomes of Patients With TBI by Timing of Extremity Fixation
eTable 6. Univariable and Multivariable Analysis for an Unfavorable Functional Outcome After 6 Months
eTable 7. Missing Variables and Multiple Imputation Model
eFigure 1. Covariate Balance Following Propensity Score Matching
eFigure 2. Distribution of Propensity Scores

This supplementary material has been provided by the authors to give readers additional information about their work.

© 2024 Zheng J et al. *JAMA Network Open*.

eTable 1. Characteristics of 120 Patients With Moderate to Severe TBI by Timing of Extremity Fixation, Unmatched Cohort

	Total (n = 120)	EEF (n = 30)	LEF (n = 90)	P	Missing (%)
**Age**, median (IQR)	35.0 (25.0-52.25)	34.5 (20.75.3-47.0)	35.5 (25.0-53.5)	.29	0
**Sex** (%)				.10	0
Female	27 (22.5)	10 (33.3)	17 (18.9)		
Male	93 (77.5)	20 (66.7)	73 (81.1)		
**Clinical care pathway** (%)				.57	0
Adimission	4 (3.3)	0	4 (4.4)		
ICU	116 (96.7)	30 (100)	86 (85.6)		
**ISS**, median (IQR)	43 (35.5-57)	43.0 (34.0.3-50.0)	44.0 (36.5-57.0)	.30	2
ISS≤ 16	3 (2.5)	1 (3.3)	2(2.3)	1	
ISS> 16	115 (97.5)	29 (96.7)	86 (97.7)		
**Upper extremity AIS** (%)				.91	0
AIS< 3	67 (55.8)	17(56.7)	50 (55.6)		
AIS≥ 3	53 (44.2)	13(43.3)	40 (44.4)		
**Lower extremity AIS** (%)				.16	0
AIS< 3	49 (40.8)	9 (30.0)	40 (44.4)		
AIS≥ 3	71 (59.2)	21 (70.0)	50 (55.)		
**GCS** (%)				.81	0
Moderate (9-12)	30 (25.0)	7 (23.3)	23 (25.6)		
Severe (3-8)	90 (75.0)	23 (76.7)	67 (74.4)		
**GCS Motor**, median(IQR)	1 (1-5)	1 (1-5)	2 (1-5)	.76	0
**Pupillary reactivity** (%)				.72	7 (5.8)
Both reactive	93 (77.5)	24 (80.0)	69 (83.1)		
One reactive	9 7.5)	2 (6.7)	7 (8.4)		
Both non-reactive	11 (9.1)	4 (13.3)	7 (5.6)		
**ASA PS** (%)				.52	4 (3.3)
Healthy	80 (66.7)	21 (70.0)	59 (65.5)		
Mild systemic disease	33 (27.5)	7 (23.3)	26 (28.8)		
Severe systemic	3 (2.5)	0	3 (2.5)		

© 2024 Zheng J et al. *JAMA Network Open.*

disease					
**Cranial surgery** (%)				.82	0
Yes	34 (28.3)	9 (30.0)	25 (73.5)		
	Total (n = 120)	EEF (n = 30)	LEF (n = 90)	P	Missing (%)
No	86 (71.7)	21 (70.0)	25 (72.2)		
**CT abnormality** (%)				.70	0
Yes	105 (87.5)	27 (90.0)	78 (86.7)		
No	13 (10.8)	3 (10.0)	10 (11.1)		
Uninterpretable	2 (1.7)	0	2 (2.2)		

Abbreviations: TBI, traumatic brain injury; EEF, early extremity fixation; LEF, late extremity fixation; IQR, interquartile range; ER, emergency room; ICU, intensive care unit; ISS, injury severity score; AIS, abbreviated injury scale; GCS, Glasgow Coma Scale; ASA PS, pre-injury American Society of Anesthesiologists Physical Status; CT, computed tomoghy.

© 2024 Zheng J et al. *JAMA Network Open*.

eTable 2. Characteristics of 58 Patients With Moderate to Severe TBI by Timing of Extremity Fixation, Matched Cohort

	EEF (n = 29)	LEF (n = 29)	SMD (before matching)	SMD (after matching)
**Age**, median (IQR)	35 (24-37)	31 (24-45)	-0.28	-0.01
**Sex** (%)			-0.23	-0.04
Female	9 (31.0)	8 (27.6)		
Male	20 (69)	21 (72.4)		
**ISS**, mean (SD)	44.6 (13.9)	44.9 (15.4)	-0.11	0.009
Lower extremity AIS (%)			-0.23	0.03
AIS<3	9 (31.0)	9(31.0)		
AIS≥3	20 (69)	20 (69)		
GCS	3 (3-8)	5 (3-7)	-0.11	0.008
Pupillary reactivity (%)				
Both reactive	23 (79.3)	25 (86.2)		
One reactive	2 (6.9)	2 (6.9)	-0.11	0.03
Both non-reactive	4 (13.8)	2 (6.9)	0.16	0.09
ASA PS (%)			-0.14	0.05
Healthy	23 (79.3)	22 (75.9)		
Mild to Severe systemic disease	6 (20.7)	7 (24.1)		
Cranial surgery			0.07	0.03
Yes	9 (31.0)	9 (31.0)		
No	20 (69)	20 (69)		
CT abnormality			0.04	-0.09
Yes	25 (86.2)	26 (89.7)		
No	4 (13.8)	3 (10.3)		

Abbreviations: TBI, traumatic brain injury; EEF, early extremity fixation; LEF, late extremity fixation; SMD, standardized mean difference; SD, standard deviation; ISS, injury severity score; AIS, abbreviated injury scale; GCS, Glasgow Coma Scale; ASA PS, pre-injury American Society of Anesthesiologists Physical Status; CT, computed tomography.

© 2024 Zheng J et al. *JAMA Network Open*.

eTable 3. Characteristics of 122 Patients With Mild TBI by Timing of Extremity Fixation, Unmatched Cohort

	Total (n = 122)	EEF (n = 39)	LEF (n = 83)	P
**Age**, median (IQR)	46.0 (29.75-61)	45 (27-69)	48 (30-61)	.83
**Sex** (%)				.14
Female	39 (32.0)	16 (41.0)	23 (27.7)	
Male	83 (68.0)	23 (59.0)	60 (72.3	
**Clinical care pathway** (%)				.20
ER	1(0.8)	1 (0.8)	0	
Adimission	4 (3.3)	10 (25.6)	30 (36.1)	
ICU	116 (96.7)	28 (71.8)	53 (63.9)	
**ISS**, median (IQR)	29 (19.75-41)	34 (22-48)	27 (19-36)	.21
ISS≤ 16	17 (13.9)	6 (15.4)	11 (13.3)	.75
ISS> 16	105 (86.1)	33 (84.6)	72 (86.7)	
**Upper extremity AIS** (%)				.58
AIS< 3	67 (54.9)	20 (51.3)	47 (56.6)	
AIS≥ 3	55 (45.1)	19 (48.7)	36 (43.4)	
**Lower extremity AIS** (%)				.49
AIS< 3	65 (53.3)	19 (48.7)	46 (55.4)	
AIS≥ 3	57 (46.7)	20 (51.3)	37 (44.6)	
**GCS Motor**, median(IQR)	6 (6-6)	6 (6-6)	6 (6-6)	.49
**Pupillary reactivity** (%)				.54
Both reactive	111 (91.0)	37(94.9)	74 (89.2)	
One reactive	2 (1.6)	1 (2.6)	1 (1.2)	
Both non-reactive	2 (1.6)	0	2(2.4)	
**ASA PS** (%)				.64
Healthy	72 (59.0)	25 (64.1)	47 (56.6)	
Mild systemic disease	43 (35.2)	12 (30.8)	31 (37.3)	
Severe systemic disease	5(4.1)	1 (2.6)	4 (4.8)	
	Total (n = 122)	EEF (n = 39)	LEF (n = 83)	P

**Cranial surgery** (%)				.59
Yes	12 (9.8)	3 (7.7)	9 (10.8)	
No	110 (90.2)	36 (92.3)	74 (89.2)	
**CT abnormality** (%)				.03
Yes	71 (58.2)	17 (43.6)	54 (65.1)	
No	49 (40.2)	22 (56.4)	27 (32.5)	
Uninterpretable	2 (1.6)	0	2 (2.4)	

Abbreviations: TBI, traumatic brain injury; EEF, early extremity fixation; LEF, late extremity fixation; IQR, interquartile range; ER, emergency room; ICU, intensive care unit; ISS, injury severity score; AIS, abbreviated injury scale; GCS, Glasgow Coma Scale; ASA PS, pre-injury American Society of Anesthesiologists Physical Status; CT, computed tomoghy.

© 2024 Zheng J et al. *JAMA Network Open.*

eTable 4. Characteristics of 70 Patients With Mild TBI by Timing of Extremity Fixation, Matched Cohort

	EEF (n = 35)	LEF (n = 35)	SMD[a] (before matching)	SMD (after matching)
**Age**, mean(SD)	35 (20.9)	34.6 (18.0)	-0.04	0.05
**Sex** (%)			-0.23	-0.05
Female	12 (34.3)	12 (34.3)		
Male	23 (65.7)	23 (65.7)		
**ISS**, mean(SD)	34.4 (16.7)	34.4 (15.3)	0.26	-0.02
Lower extremity AIS (%)			-0.14	0.05
AIS<3	18 (51.4)	19 (54.3)		
AIS≥3	17 (48.6)	16 (45.7)		
ASA PS (%)			-0.13	0.02
Healthy	22 (62.9)	22 (62.9)		
Mild to Severe systemic disease	13 (37.1)	13 (37.1)		
CT abnormality				
Yes	17 (48.6)	18 (51.4)	-0.46	-0.06
No	18 (51.4)	17 (48.6)		

[a]Patients with mild traumatic brain injuries often have similar Glasgow Coma Scale scores and are less likely to require cranial surgery. Additionally, abnormal pupillary reactivity is not common in these patients. Therefore, we did not include these three variables in the logistic model for propensity score matching.

Abbreviations: TBI, traumatic brain injury; EEF, early extremity fixation; LEF, late extremity fixation; SMD, standardized mean difference; SD, standard deviation; ISS, injury severity score; AIS, abbreviated injury scale; GCS, Glasgow Coma Scale; ASA PS, pre-injury American Society of Anesthesiologists Physical Status; CT, computed tomography.

© 2024 Zheng J et al. *JAMA Network Open*.

eTable 5. Secondary Outcomes of Patients With TBI by Timing of Extremity Fixation

	All severity TBI			Moderate-severe TBI			Mild TBI		
	EEF (n = 69)	LEF (n = 69)	P	EEF (n = 29)	LEF (n = 29)	P	EEF (n = 35)	LEF (n = 35)	P
In-hospital mortality (%)	3 (4.3)	0	0.25	2 (6.9)	1 (3.4)	.61	1 (2.9)	0	1
30-d mortality (%)	3 (4.3)	0	0.25	2(6.9)	1 (3.4)	.61	1 (2.9)	0	1
6-m mortality (%)	3 (4.3)	3 (4.3)	0.98	2 (6.9)	1 (3.4)	.61	1 (2.9)	1 (2.9)	1
Complications									
Respiratory (%)	11 (15.9)	17 (24.6)	0.28	7 (24.1)	9 (31.0)	.62	6 (17.1)	4 (11.4)	.54
Cardiac (%)	2 (2.9)	6 (8.7)	0.18	2 (6.9)	2 (6.9)	1	1 (2.9)	3 (8.6)	.62
Raised ICP (%)	10 (14.5)	9 (13.0)	0.99	7 (24.1)	5 (17.2)	.57	2 (5.4)	3 (8.6)	1
UTI (%)	4 (5.8)	6 (8.7)	0.52	3 (10.3)	6 (20.7)	.22	1 (2.9)	3 (8.6)	.62
Metabolic (%)	2 (2.9)	6 (8.7)	0.24	3 (10.3)	2 (6.9)	.88	0	1 (2.9)	1
Delayed hematoma (%)	5 (7.2)	2 (2.9)	0.41	3 (10.3)	0	.24	2 (5.7)	0	.49
Seizures (%)	2 (2.9)	4 (5.8)	0.57	2 (6.9)	1 (3.4)	.56	1 (2.9)	0	1
DVT (%)	3 (4.3)	4 (5.8)	0.84	1 (3.4)	1 (3.4)	1	1 (2.9)	1 (2.9)	1
LOS, median (IQR)	20.0 (12.3-35.8)	24.60 (13.6-41.6)	0.18	26.4 (15-42)	30 (17.2-48.2)	.61	16.2 (7.0-26.4)	21.1 (9.6-32.5)	.22

Abbreviations: TBI, traumatic brain injury; EEF, early extremity fixation; LEF, late extremity fixation; ICP, intracranial pressure; UTI, urinary tract infection; DVT, deep vein thrombosis; LOS, length of stay; IQR, interquartile range.

© 2024 Zheng J et al. *JAMA Network Open.*

eTable 6. Univariable and Multivariable Analysis for an Unfavorable Functional Outcome After 6 Months

	Univariable analysis		Multivariable analysis	
	OR (95% CI)	P	OR (95% CI)	P
Age	1.01 (1.00-1.03)	.12	1.02(1.00-1.04)	.07
Female	1.08 (0.60-2.00)	.80	1.10 (0.53-2.29)	.80
GCS: 3-12	0.93 (0.88-0.98)	.006[a]	1.07 (0.65-1.76)	.81
ISS	1.03 (1.01-1.05)	.001[a]	1.02 (0.99-1.04)	.17
Pupils both non-reactive	3.54 (1.75-7.16)	.001[a]	18.75 (2.06-170.85)	.01[a]
Lower extremity AIS ≥ 3	2.62 (1.49-4.60)	.001[a]	3.43 (1.66-7.00)	.001[a]
EEF	1.13 (0.64-1.98)	.68	1.14 (0.58-2.25)	.70
GCS motor	0.83 (0.73-0.93)	.002[a]	0.83 (0.60-1.18)	.29
Mild to severe systemic disease	1.87 (1.08-3.24)	.03[a]	1.90 (0.92-3.92)	.08
Cranial surgery	1.93 (1.01-3.67)	.05[a]	1.78 (0.81-3.90)	.15

© 2024 Zheng J et al. *JAMA Network Open.*

Abnormal CT	1.95 (1.03-3.73)	.04[a]	0.65 (0.50-8.44)	.74
Upper extremity AIS ≥ 3	0.98 (0.56-1.74)	.95		

a: p values < .05

Abbreviations: OR, odds ratio; CI, late extremity fixation; GCS, Glasgow Coma Scale; ISS, injury severity score; AIS, abbreviated injury scale; EEF, early extremity fixation; ASA PS, pre-injury American Society of Anesthesiologists Physical Status; CT, computed tomography.

© 2024 Zheng J et al. *JAMA Network Open.*

eTable 7. Missing Variables and Multiple Imputation Model

	Missing, n (%)	Multiple imputation model
Total ISS	4 (1.6)	Linear regression
GCS	11 (4.3)	Logistics regression
GCS motor	4 (1.6)	Logistics regression
Pupils	17 (6.7)	Logistics regression
ASA PS	8 (3.2)	Logistics regression
GOSE at 6 months	18 (7.1)	Logistics regression

Abbreviations: ISS, injury severity score; GCS, Glasgow Coma Scale; ASA PS, pre-injury American Society of Anesthesiologists Physical Status; GOSE, Glasgow Outcome Scale Extended.

© 2024 Zheng J et al. *JAMA Network Open.*

eFigure 1. Covariate Balance Following Propensity Score Matching

A. Covariate balance following propensity score matching in all patients with TBI

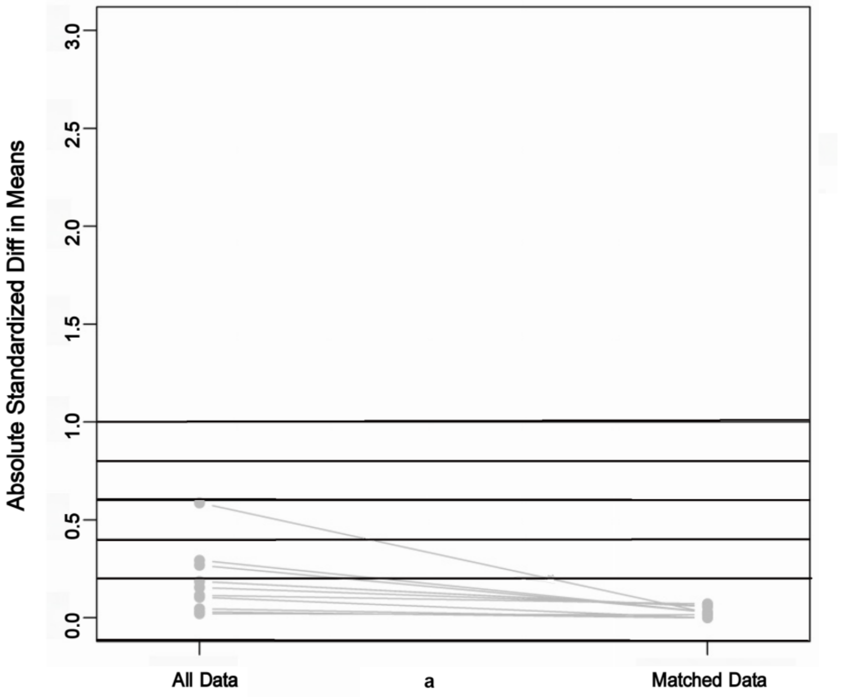

B. Covariate balance following propensity score matching in moderate-severe TBI patients subgroup

© 2024 Zheng J et al. *JAMA Network Open.*

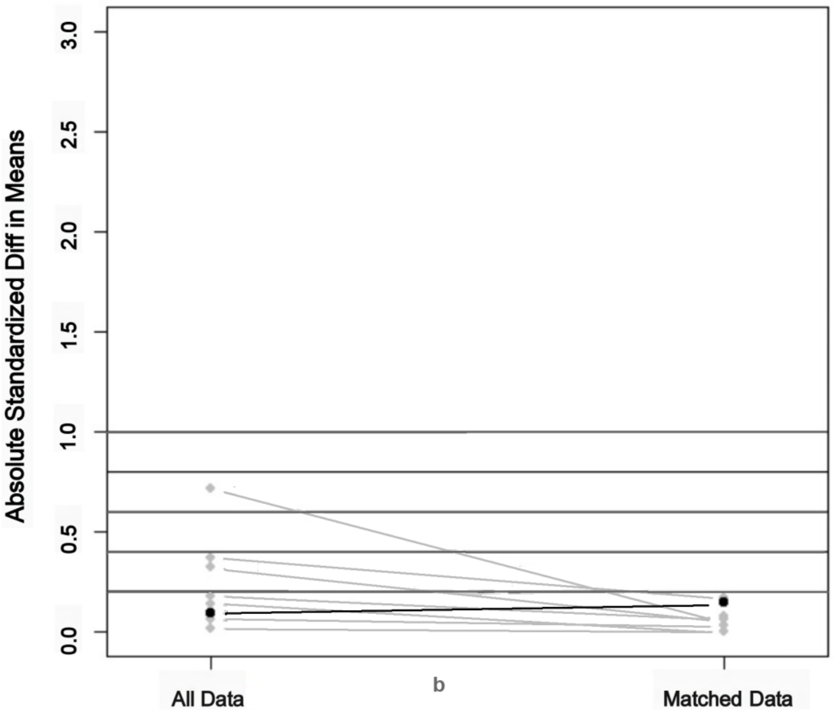

© 2024 Zheng J et al. *JAMA Network Open*.

C. Covariate balance following propensity score matching in mild TBI patients subgroup

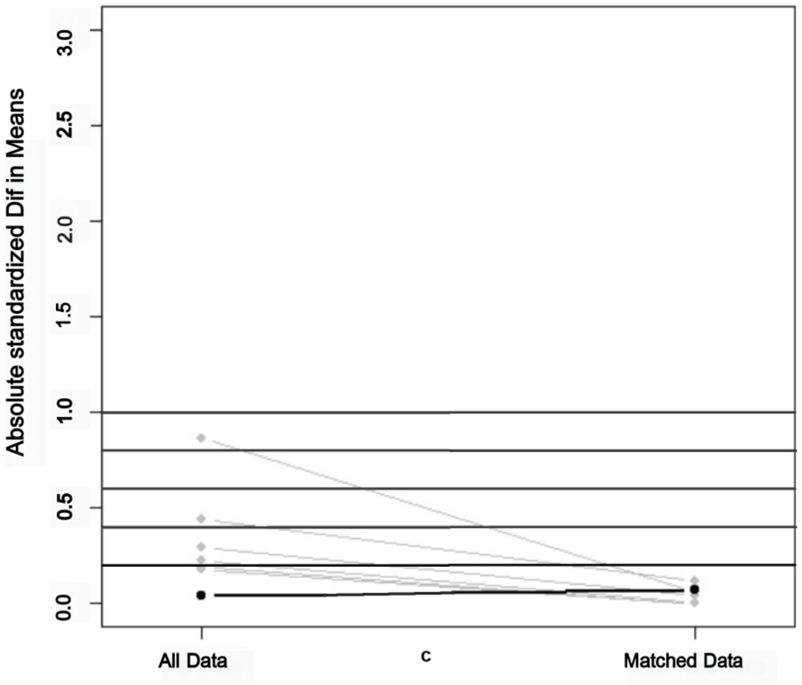

© 2024 Zheng J et al. *JAMA Network Open.*

eFigure 2. Distribution of Propensity Scores

A. Distribution of Propensity Scores in all patients with TBI

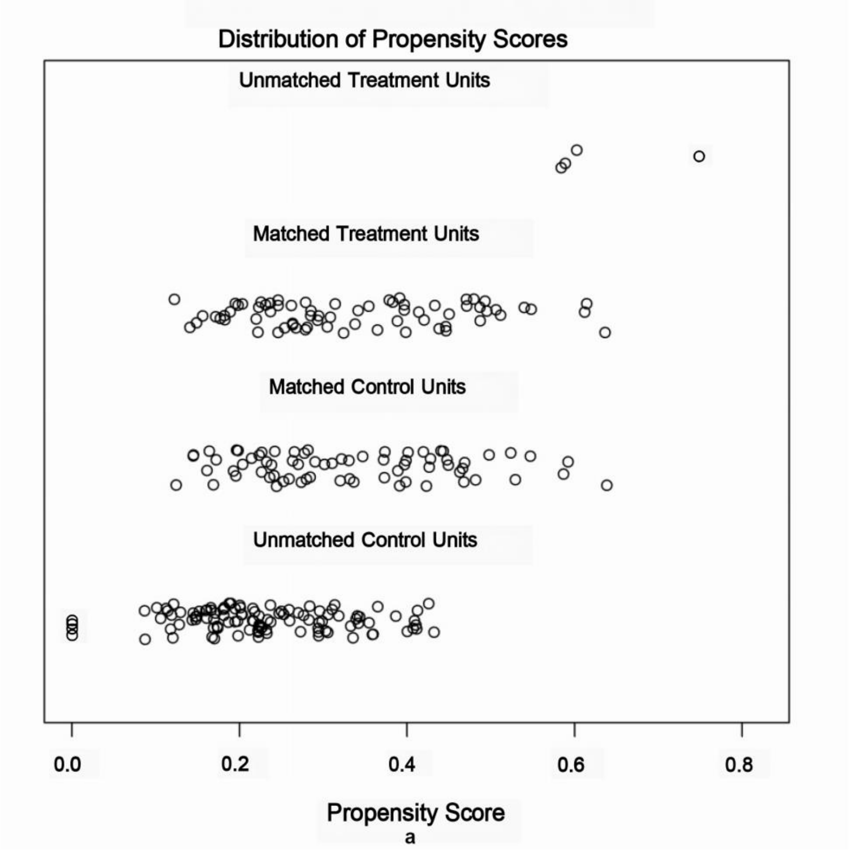

B. Distribution of Propensity Scores in moderate-severe TBI patients subgroup

© 2024 Zheng J et al. *JAMA Network Open.*

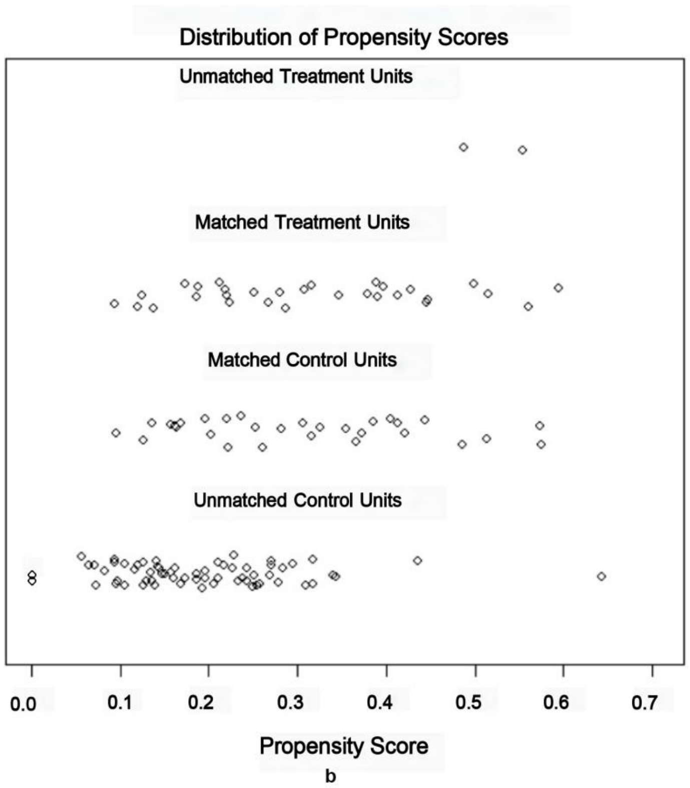

C. Distribution of Propensity Scores in mild TBI patients subgroup

© 2024 Zheng J et al. *JAMA Network Open.*

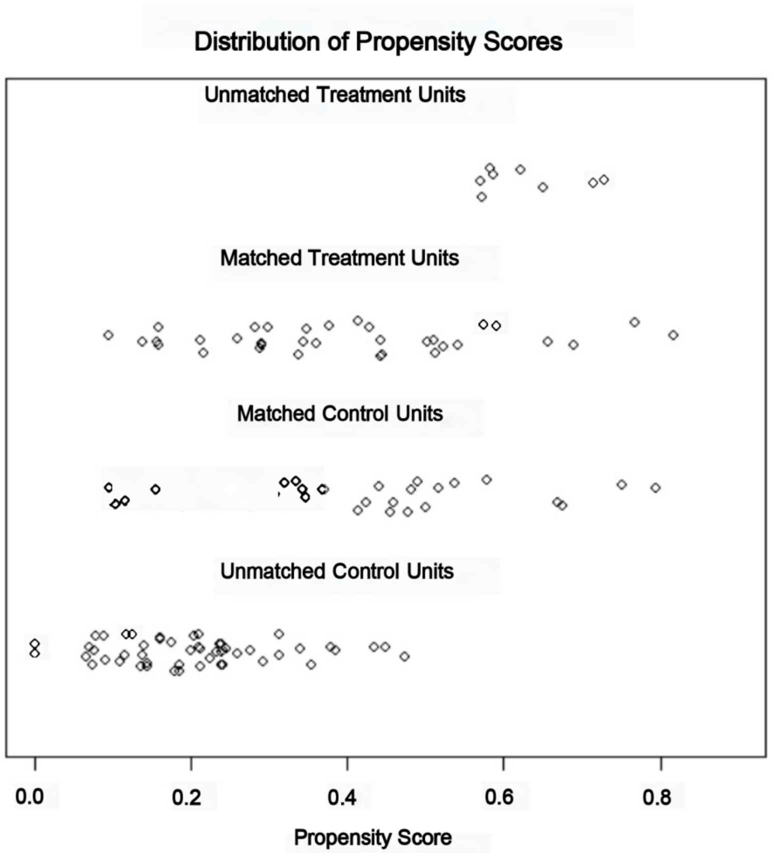

C

© 2024 Zheng J et al. *JAMA Network Open*.
