# Supplementary material for: Early vs Late Fixation of Extremity Fractures Among Adults With Traumatic Brain Injury
Source: JAMA Netw Open. 2024 Mar 8;7(3):e241556. doi: 10.1001/jamanetworkopen.2024.1556 (PMC10924246; doi:10.1001/jamanetworkopen.2024.1556)
Supplement: Supplement 2. — Nonauthor Contributors [file jamanetwopen-e241556-s002.pdf]

*Group Name(s): Collaborative European NeuroTrauma Effectiveness Research in Traumatic Brain Injury (CENTER-TBI) Investigators and								
*First Name and Middle Initial(s)	*Last Name	*Suffix (eg, Jr, III)	Academic Degrees	Institution	Location (city, state/province, country)	Role or Contribution, eg, chair, principal investigator	Group (if more than 1 Group listed in the byline) and/or Subgroup (eg, Steering Committee)	
Jonathan	Rosand			Broad Institute, Cambridge MA Ha	Boston MA, USA	CENTER-TBI Associated Participant		
Marta	Correia			Radiology/MRI department, MRC	Cambridge, UK	CENTER-TBI Associated Participant		
Pablo	Gagliardo			Fundación Instituto Valenciano de	Jerusalem, Israel	CENTER-TBI Associated Participant		
Paul M.	Vespa			Director of Neurocritical Care, Un	Los Angeles, USA	CENTER-TBI Associated Participant		
Robert	Stevens			Division of Neuroscience Critical	Baltimore, USA	CENTER-TBI Associated Participant		
Alex	Furmanov			Department of Neurosurgery, Had	Jerusalem, Israel	CENTER-TBI Investigator		
Alexandra	Brazinova			Department of Public Health, Fac	Trnava, Slovakia	CENTER-TBI Participant		
Alexandre	Ghuysen			Emergency Department, CHU	Liège, Belgium	CENTER-TBI Investigator		
Alfonso	Lagares			Department of Neurosurgery, Hos	Madrid, Spain	CENTER-TBI Investigator		
Ana	Kowark			Department of Anaesthesiology, U	Aachen, Germany	CENTER-TBI Investigator		
Ana M.	Castaño-León			Department of Neurosurgery, Hos	Madrid, Spain	CENTER-TBI Investigator		
Ancuta	Negru			Department of Neurosurgery, Eme	Timisoara, Romania	CENTER-TBI Investigator		
Andreea	Radoi			Neurotraumatology and Neurosur	Barcelona, Spain	CENTER-TBI Investigator		
Anna	Piippo-Karjalainen			Department of Neurosurgery, Hels	Helsinki, Finland	CENTER-TBI Investigator		
Anna	Antoni			Trauma Surgery, Medical Univers	Vienna, Austria	CENTER-TBI Investigator		
Antonio	Belli			NIHR Surgical Reconstruction and	Birmingham, UK	CENTER-TBI Investigator		
Arminas	Ragauskas			Department of Neurosurgery, Kau	Vilnius, Lithuania	CENTER-TBI Investigator		
Arturo	Chieregato			NeuroIntensive Care, Niguarda H	Milan, Italy	CENTER-TBI Investigator		
Aurelie	Lejeune			Department of Anesthesiology-Int	Lille, France	CENTER-TBI Investigator		
Bart	Depreitere			Department of Neurosurgery, Univ	Leuven, Belgium	CENTER-TBI Investigator		
Bo-Michael	Bellander			Department of Neurosurgery & An	Stockholm, Sweden	CENTER-TBI Investigator		
Bram	Jacobs			Department of Neurology, Univers	Groningen, Netherlands	CENTER-TBI Investigator		
Camilla	Brorsson			Department of Surgery and Periop	Umeå, Sweden	CENTER-TBI Investigator		
Catherine	McMahon			Department of Neurosurgery, The	Liverpool, UK	CENTER-TBI Investigator		
Christina	Rosenlund			Department of Neurosurgery, Ode	Melbourne, Victoria, Aus	CENTER-TBI Investigator		
Christos	Tolias			Department of Neurosurgery, King	London, UK	CENTER-TBI Investigator		
Claire	Dahyot-Fizelier			Intensive Care Unit, CHU Poitiers	Potiers, France	CENTER-TBI Investigator		
Costanza	Martino			Department of Anesthesia & Inter	Cesena, Italy	CENTER-TBI Investigator		
Cristina Maria	Tudora			Department of Neurosurgery, Eme	Timisoara, Romania	CENTER-TBI Investigator		
Daniel	Kondziella			Departments of Neurology, Clinica	Copenhagen, Denmark	CENTER-TBI Investigator		

Supplemental Online Content: Nonauthor Collaborators

*First name, last name, and suffix (if applicable) are required and will appear in PubMed.

***First Name and Middle Initial(s)**	***Last Name**	***Suffix (eg, Jr, III)**	Academic Degrees	Institution	Location (city, state/province, country)	Role or Contribution, eg, chair, principal investigator	Group (if more than 1 Group listed in the byline) and/or Subgroup (eg, Steering Committee)	
Đula	Đilvesi			Department of Neurosurgery, Clin	Novi Sad, Serbia	CENTER-TBI Investigator		
Egils	Valeinis			Neurosurgery clinic, Pauls Stradir	Riga, Latvia	CENTER-TBI Investigator		
Elisabeth	Schwendenwein			Trauma Surgery, Medical Univers	Vienna, Austria	CENTER-TBI Investigator		
Emmanuel	Vega			Department of Anesthesiology-Int	Lille, France	CENTER-TBI Investigator		
Faye	Johnson			Salford Royal Hospital NHS Foun	Salford, UK	CENTER-TBI Investigator		
Francesca	Grossi			Department of Anesthesia & Inter	Novara, Italy	CENTER-TBI Investigator		
Francesco	Della Corte			Department of Anesthesia & Inter	Novara, Italy	CENTER-TBI Investigator		
Gérard	Audibert			Department of Anesthesiology & I	Nancy, France	CENTER-TBI Investigator		
Giorgio	Chevallard			NeuroIntensive Care, Niguarda H	Milan, Italy	CENTER-TBI Investigator		
Guillermo	Carbayo Lozano			Department of Neurosurgery, Hos	Bilbao, Spain	CENTER-TBI Investigator		
Guus	Schoonman			Department of Neurology, Elisabe	Tilburg, the Netherlands	CENTER-TBI Investigator		
Guy	Rosenthal			Department of Neurosurgery, Hac	Jerusalem, Israel	CENTER-TBI Investigator		
Guy-Loup	Dulière			Intensive Care Unit, CHR Citadell	Liège, Belgium	CENTER-TBI Investigator		
Hans	Clusmann			Department of Neurosurgery, Mec	Aachen, Germany	CENTER-TBI Investigator		
Horia	Ples			Department of Neurosurgery, Eme	Timisoara, Romania	CENTER-TBI Investigator		
Hugo	den Boogert			Department of Neurosurgery, Rad	Nijmegen, The Netherlar	CENTER-TBI Investigator		
Hugues	Maréchal			Intensive Care Unit, CHR Citadell	Liège, Belgium	CENTER-TBI Investigator		
Iain	Haitsma			Department of Neurosurgery, Era	Rotterdam, Netherlands	CENTER-TBI Investigator		
Jagoš	Golubovic			Department of Neurosurgery, Clin	Novi Sad, Serbia	CENTER-TBI Investigator		
Jean-François	Payen			Department of Anesthesiology & I	Grenoble, France	CENTER-TBI Investigator		
Jonathan	Rhodes			Department of Anaesthesia, Critic	Edinburgh, UK	CENTER-TBI Investigator		
Joukje	van der Naalt			Department of Neurology, Univers	Groningen, Netherlands	CENTER-TBI Investigator		
Juan	Sahuquillo			Neurotraumatology and Neurosur	Barcelona, Spain	CENTER-TBI Investigator		
Lars-Owe	Koskinen			Department of Clinical Neuroscie	Umeå, Sweden	CENTER-TBI Investigator		
Lelde	Giga			Neurosurgery clinic, Pauls Stradir	Riga, Latvia	CENTER-TBI Investigator		
Leon	Levi			Department of Neurosurgery, Rar	Haifa, Israel	CENTER-TBI Investigator		
Luigi	Beretta			Department of Anesthesiology & I	Milan, Italy	CENTER-TBI Investigator		
Malinka	Rambadagalla			Department of Neurosurgery, Rez	Rezekne， Latvia	CENTER-TBI Investigator		
Maria Luisa	Azzolini			Department of Anesthesiology & I	Milan, Italy	CENTER-TBI Investigator		
Maria Rosa	Calvi			Department of Anesthesiology & I	Milan, Italy	CENTER-TBI Investigator		
Mark Steven	Coburn			Department of Anesthesiology an	Bonn, Germany	CENTER-TBI Investigator		
Matt	Thomas			Intensive Care Unit, Southmead H	Bristol, Bristol, UK	CENTER-TBI Investigator		
Mladen	Karan			Department of Neurosurgery, Clin	Novi Sad, Serbia	CENTER-TBI Investigator		

***First Name and Middle Initial(s)**	***Last Name**	***Suffix (eg, Jr, III)**	Academic Degrees	Institution	Location (city, state/province, country)	Role or Contribution, eg, chair, principal investigator	Group (if more than 1 Group listed in the byline) and/or Subgroup (eg, Steering Committee)	
Nina	Sundström			Department of Radiation Science	Umeå, Sweden	CENTER-TBI Investigator		
Pál	Barzó			Department of Neurosurgery, Uni	Szeged, Hungary	CENTER-TBI Investigator		
Paolo	Persona			Department of Anesthesia & Inter	Padova, Italy	CENTER-TBI Investigator		
Paul	Dark			University of Manchester NIHR Bi	Salford, UK	CENTER-TBI Investigator		
Pedro A.	Gomez			Department of Neurosurgery, Hos	Madrid, Spain	CENTER-TBI Investigator		
Petar	Vulekovic			Department of Neurosurgery, Clin	Novi Sad, Serbia	CENTER-TBI Investigator		
Peter	Vajkoczy			Neurologie, Neurochirurgie und P	Berlin, Germany	CENTER-TBI Investigator		
Rahul	Raj			Department of Neurosurgery, Hel	Helsinki, Finland	CENTER-TBI Investigator		
Raimund	Helbok			Department of Neurology, Neurol	Innsbruck, Austria	CENTER-TBI Investigator		
Rimantas	Vilcinis			Department of Neurosurgery, Kau	Kaunas, Lithuania	CENTER-TBI Investigator		
Roger	Lightfoot			Department of Anesthesiology & I	Southhampton, UK	CENTER-TBI Investigator		
Ronald	Bartels			Department of Neurosurgery, Rac	Nijmegen, Netherlands	CENTER-TBI Investigator		
Ronny	Beer			Department of Neurology, Neurol	Innsbruck, Austria	CENTER-TBI Investigator		
Sandra	Rossi			Department of Anesthesia & Inter	Padova, Italy	CENTER-TBI Investigator		
Saulius	Rocka			Department of Neurosurgery, Kau	Vilnius, Lithuania	CENTER-TBI Investigator		
Shirin	Frisvold			Department of Anesthesiology an	Tromso, Norway	CENTER-TBI Investigator		
Simona	Cavallo			Department of Anesthesia & ICU,	Torino, Italy	CENTER-TBI Investigator		
Stefan	Jankowski			Neurointensive Care , Sheffield T	Sheffield, UK	CENTER-TBI Investigator		
Tomas	Tamosuitis			Department of Neurosurgery, Kau	Kaunas, Lithuania	CENTER-TBI Investigator		
Toril	Skandsen			1. Department of Neuromedicine and Movement Science, Norwegian University of Science and Technology, NTNU 2. Department of Physical Medicine and Rehabilitation, St.Olavs Hospital, Trondheim University Hospital	1.Trondheim, Norway; 2.Trondheim, Norway	1. CENTER-TBI Investigator; 2. CENTER-TBI Investigator		
Veronika	Zelinkova			Department of Public Health, Fac	Trnava, Slovakia	CENTER-TBI Investigator		
Zoltán	Vámos			Department of Anaesthesiology a	Pécs, Hungary	CENTER-TBI Participant + Investigator		
Alice	Theadom			National Institute for Stroke and A	Auckland, New Zealand	CENTER-TBI Participant		
Amra	Čović			Institute of Medical Psychology ar	Göttingen, Germany	CENTER-TBI Participant		

*First name, last name, and suffix (if applicable) are required and will appear in PubMed.

*First Name and Middle Initial(s)	*Last Name	*Suffix (eg, Jr, III)	Academic Degrees	Institution	Location (city, state/province, country)	Role or Contribution, eg, chair, principal investigator	Group (if more than 1 Group listed in the byline) and/or Subgroup (eg, Steering Committee)	
Ana	Mikolic			Department of Public Health, Eras	Rotterdam, Netherlands	CENTER-TBI Participant		
Béla	Melegh			Department of Medical Genetics,	Pécs, Hungary	CENTER-TBI Participant		
Ben	Glocker			Department of Computing, Imperi	London, UK	CENTER-TBI Participant		
Benjamin	Gravesteijn			Department of Public Health, Eras	Rotterdam, Netherlands	CENTER-TBI Participant		
Braden Te	Ao			National Institute for Stroke and A	Auckland, New Zealand	CENTER-TBI Participant		
Caroline	van Heugten			Movement Science Group, Facult	Oxford, UK	CENTER-TBI Participant		
Charlie	Sewalt			Department of Public Health, Eras	Rotterdam, Netherlands	CENTER-TBI Participant		
Daan	Nieboer			Department of Public Health, Eras	Rotterdam, Netherlands	CENTER-TBI Participant		
Dana	Pisica			Department of Public Health, Eras	Rotterdam, Netherlands	CENTER-TBI Participant		
Daniel	Rueckert			Department of Computing, Imperi	London, UK	CENTER-TBI Participant		
Daphne	Voormolen			Department of Public Health, Eras	Rotterdam, Netherlands	CENTER-TBI Participant		
Dick	Tibboel			Intensive Care and Department o	Rotterdam, Netherlands	CENTER-TBI Participant		
Emma	Donoghue			Australian & New Zealand Intensi	Melbourne, Australia	CENTER-TBI Participant		
Emmanuel	Stamatakis			Division of Anaesthesia, Universit	Cambridge, UK	CENTER-TBI Participant		
Ernest	van Veen			Department of Public Health, Eras	Rotterdam,Netherlands	CENTER-TBI Participant		
Erwin	Kompanje			Department of Intensive Care and	Rotterdam, Netherlands	CENTER-TBI Participant		
Eveline	Wiegers			Department of Public Health, Eras	Rotterdam, Netherlands	CENTER-TBI Participant		
Frederick A.	Zeiler			1.Division of Anaesthesia, University of Cambridge, Addenbrooke’s Hospital; 2. Section of Neurosurgery, Department of Surgery, Rady Faculty of Health Sciences, University of Manitoba	1. Cambridge, UK ; 2. Winnipeg, MB, Canada	1. CENTER-TBI Participant; 2. CENTER-TBI Participant		
Geoffrey	Manley			Department of Neurological Surge	San Francisco, Californi	CENTER-TBI Participant		
Gregory	Van der Steen			Department of Neurosurgery, Ant	Edegem, Belgium	CENTER-TBI Participant		
Guoyi	Gao			Department of Neurosurgery, Sha	Shanghai, China	CENTER-TBI Participant		
Guy	Williams			Division of Anaesthesia, Universit	Cambridge, UK	CENTER-TBI Participant		
Helen	Dawes			Movement Science Group, Facult	Oxford, UK	CENTER-TBI Participant		
Isabel Retel	Helmrich			Department of Public Health, Eras	Rotterdam, Netherlands	CENTER-TBI Participant		
Jan	Verheyden			Institute of Medical Psychology ar	Göttingen, Germany	CENTER-TBI Participant		
Janos	Sandor			Division of Biostatistics and Epide	Debrecen, Hungary	CENTER-TBI Participant		
Jeffrey V.	Rosenfeld			National Trauma Research Institu	Boston MA, USA	CENTER-TBI Participant		

*First name, last name, and suffix (if applicable) are required and will appear in PubMed.

*First Name and Middle Initial(s)	*Last Name	*Suffix (eg, Jr, III)	Academic Degrees	Institution	Location (city, state/province, country)	Role or Contribution, eg, chair, principal investigator	Group (if more than 1 Group listed in the byline) and/or Subgroup (eg, Steering Committee)	
Jilske	Huijben			Department of Public Health, Eras	Rotterdam, Netherlands	CENTER-TBI Participant		
Ji-yao	Jiang			Department of Neurosurgery, Sha	Shanghai, China	CENTER-TBI Participant		
Joanne	Brooker			Australian & New Zealand Intensi	Melbourne, Australia	CENTER-TBI Participant		
Johannes	Gratz			Department of Anesthesia, Critica	Vienna, Austria	CENTER-TBI Participant		
Juanita A.	Haagsma			Department of Public Health, Eras	Rotterdam, Netherlands	CENTER-TBI Participant		
Kelly	Jones			National Institute for Stroke and A	Auckland, New Zealand	CENTER-TBI Participant		
Kevin K.W.	Wang			Department of Emergency Medici	Gainesville, Florida, US/	CENTER-TBI Participant		
Kimberley	Velt			Department of Public Health, Eras	Rotterdam, Netherlands	CENTER-TBI Participant		
Lindsay	Horton			Division of Psychology, University	Stirling, UK	CENTER-TBI Participant		
Lindsay	Wilson			Division of Psychology, Univer	Stirling, UK	CENTER-TBI Participant		
Marc	Maegele			Cologne-Merheim Medical Center	Cologne, Germany	CENTER-TBI Participant		
Marek	Majdan			Department of Public Health, Fac	Trnava, Slovakia	CENTER-TBI Participant		
Marjolein	Timmers			Department of Intensive Care and	Rotterdam, Netherlands	CENTER-TBI Participant		
Mark Steven	Taylor			Department of Public Health, Fac	Trnava, Slovakia	CENTER-TBI Participant		
Martin	Rusnák			International Neurotrauma Resea	Vienna, Austria	CENTER-TBI Participant		
Matej	Oresic			School of Medical Sciences, Öreb	Örebro, Sweden	CENTER-TBI Participant		
Mathieu	van der Jagt			Department of Intensive Care Adu	Rotterdam, Netherlands	CENTER-TBI Participant		
Matti	Pirinen			Institute for Molecular Medicine F	Helsinki, Finland	CENTER-TBI Participant		
Mike	Jarrett			Quesgen Systems Inc.	Burlingame, California, U	CENTER-TBI Participant		
Nadine	Schäfer			Institute of Research in Operative	Cologne, Germany	CENTER-TBI Participant		
Natascha	Perera			International Projects Managemen	Munchen, Germany	CENTER-TBI Participant		
Nicola	Curry			Oxford University Hospitals NHS T	Oxford, UK	CENTER-TBI Participant		
Nicole	von Steinbüchel			Institute of Medical Psychology ar	Göttingen, Germany	CENTER-TBI Participant		
Patrick	Esser			Movement Science Group, Facult	Oxford, UK	CENTER-TBI Participant		
Peter	Bragge			BehaviourWorks Australia, Monas	Melbourne, Australia	CENTER-TBI Participant		
Peter	Cameron			ANZIC Research Centre, Monash	Melbourne, Victoria, Aus	CENTER-TBI Participant		
Peter	Ylén			VTT Technical Research Centre	Tampere, Finland	CENTER-TBI Participant		
Philippe	Azouvi			Raymond Poincare hospital, Assis	Paris, France	CENTER-TBI Participant		
Pradeep	George			Karolinska Institutet, INCF Interna	Stockholm, Sweden	CENTER-TBI Participant		
Rolf	Lefering			Institute of Research in Operative	Cologne, Germany	CENTER-TBI Participant		
Romuald	Beauvais			International Projects Managemen	Munchen, Germany	CENTER-TBI Participant		
Samuli	Ripatti			Institute for Molecular Medicine	Helsinki, Finland	CENTER-TBI Participant		
Simon	Stanworth			Oxford University Hospitals NHS T	Oxford, UK	CENTER-TBI Participant		

Supplemental Online Content: Nonauthor Collaborators
*First name, last name, and suffix (if applicable) are required and will appear in PubMed.

*First Name and Middle Initial(s)	*Last Name	*Suffix (eg, Jr, III)	Academic Degrees	Institution	Location (city, state/province, country)	Role or Contribution, eg, chair, principal investigator	Group (if more than 1 Group listed in the byline) and/or Subgroup (eg, Steering Committee)	
Suzanne	Polinder			Department of Public Health, Eras	Rotterdam, Netherlands	CENTER-TBI Participant		
Sylvia	Richardson			Director, MRC Biostatistics Unit, C	Cambridge, UK	CENTER-TBI Participant		
Valery L.	Feigin			National Institute for Stroke and A	Auckland, New Zealand	CENTER-TBI Participant		
Veronika	Rehorčíková			Department of Public Health, Fac	Trnava, Slovakia	CENTER-TBI Participant		
Vibeke	Brinck			Quesgen Systems Inc.	Burlingame, California, U	CENTER-TBI Participant		
William	Stewart			Department of Neuropathology, G	Glasgow, UK	CENTER-TBI Participant		
Wim	Van Hecke			icoMetrix NV	Leuven, Belgium	CENTER-TBI Participant		
Zhihui	Yang			Broad Institute, Cambridge MA Ha	Boston MA, USA	CENTER-TBI Participant		
Abayomi	Sorinola			Department of Neurosurgery, Univ	Pécs, Hungary	CENTER-TBI Participant + Investigator		
Abhishek	Dixit			Division of Anaesthesia, Universit	Cambridge, UK	CENTER-TBI Participant + Investigator		
Alessia	Vargiolu			NeuroIntensive Care Unit, Depart	Monza, Italy	CENTER-TBI Participant + Investigator		
Alessio	Caccioppola			Neuro ICU, Fondazione IRCCS C	Milan, Italy	CENTER-TBI Participant + Investigator		
Andras	Buki			Department of Neurosurgery, Med	Pécs, Hungary	CENTER-TBI Participant + Investigator		
Andreas	Unterberg			Department of Neurosurgery, Univ	Heidelberg, Germany	CENTER-TBI Participant + Investigator		
Angelos G.	Kolias			Division of Neurosurgery, Departr	Cambridge, UK	CENTER-TBI Participant + Investigator		
Ari	Ercole			Division of Anaesthesia, Universit	Cambridge, UK	CENTER-TBI Participant + Investigator		
Aurore	Thibaut			Cyclotron Research Center , Univ	Liège, Belgium	CENTER-TBI Participant + Investigator		
Benoit	Misset			Cyclotron Research Center , Univ	Liège, Belgium	CENTER-TBI Participant + Investigator		
Cecilie	Roe			Department of Physical Medici	Oslo, Norway	CENTER-TBI Participant + Investigator		
Damien	Galanaud			Anesthesie-Réanimation, Assista	Paris, France	CENTER-TBI Participant + Investigator		
Daniel	Whitehouse			Division of Anaesthesia, Universit	Cambridge, UK	CENTER-TBI Participant + Investigator		
David	Nelson			Department of Physiology and Ph	Stockholm, Sweden	CENTER-TBI Participant + Investigator		
Didier	Ledoux			Cyclotron Research Center , Univ	Liège, Belgium	CENTER-TBI Participant + Investigator		
Dominique	Van Praag			Psychology Department, Antwerp	Edegem, Belgium	CENTER-TBI Participant + Investigator		
Eirik	Helseth			Department of Neurosurgery, Osl	Oslo, Norway	CENTER-TBI Participant + Investigator		
Emiliana	Calappi			Neuro ICU, Fondazione IRCCS C	Milan, Italy	CENTER-TBI Participant + Investigator		
Endre	Czeiter			Department of Neurosurgery, Med	Pécs, Hungary	CENTER-TBI Participant + Investigator		
Erzsébet	Ezer			Department of Anaesthesiology a	Pécs, Hungary	CENTER-TBI Participant + Investigator		
Evgenios	Kornaropoulos			Division of Anaesthesia, Universit	Cambridge, UK	CENTER-TBI Participant + Investigator		
Fabrizio	Ortolano			Neuro ICU, Fondazione IRCCS C	Milan, Italy	CENTER-TBI Participant + Investigator		
Habib	Benali			Anesthesie-Réanimation, Assista	Paris, France	CENTER-TBI Participant + Investigator		
Hadie	Adams			Division of Neurosurgery, Departr	Cambridge, UK	CENTER-TBI Participant + Investigator		

Supplemental Online Content: Nonauthor Collaborators
*First name, last name, and suffix (if applicable) are required and will appear in PubMed.

*First Name and Middle Initial(s)	*Last Name	*Suffix (eg, Jr, III)	Academic Degrees	Institution	Location (city, state/province, country)	Role or Contribution, eg, chair, principal investigator	Group (if more than 1 Group listed in the byline) and/or Subgroup (eg, Steering Committee)	
Inge A.M.	van Erp			Dept. of Neurosurgery, Leiden Ur	The Hague, The Netherl	CENTER-TBI Participant + Investigator		
Jamie D.	Cooper			School of Public Health & PM, Mc	Melbourne, Victoria, Aus	CENTER-TBI Participant + Investigator		
Jens	Dreier			Center for Stroke Research Berlir	Berlin, Germany	CENTER-TBI Participant + Investigator		
Jeroen T.J.M.	van Dijck			Dept. of Neurosurgery, Leiden Ur	The Hague, The Netherl	CENTER-TBI Participant + Investigator		
Jonathan	Coles			Department of Anesthesia & Neur	Cambridge, UK	CENTER-TBI Participant + Investigator		
József	Nyirádi			János Szentágothai Research Ce	Pécs, Hungary	CENTER-TBI Participant + Investigator		
Jussi P.	Posti			Division of Clinical Neurosciences	Turku, Finland	CENTER-TBI Participant + Investigator		
Kelly	Foks			Department of Neurology, Erasmu	Rotterdam, Netherlands	CENTER-TBI Participant + Investigator		
Linda	Lanyon			Karolinska Institutet, INCF Interna	Stockholm, Sweden	CENTER-TBI Participant + Investigator		
Louis	Puybasset			Department of Anesthesiology an	Paris, France	CENTER-TBI Participant + Investigator		
Marco	Carbonara			Neuro ICU, Fondazione IRCCS C	Milan, Italy	CENTER-TBI Participant + Investigator		
Marek	Czosnyka			Brain Physics Lab, Division of Ne	Cambridge, UK	CENTER-TBI Participant + Investigator		
Martin	Fabricius			Departments of Neurology, Clinica	Copenhagen, Denmark	CENTER-TBI Participant + Investigator		
Morten	Blaabjerg			Department of Neurology, Odense	Odense, Denmark	CENTER-TBI Participant + Investigator		
Noémi	Kovács			Hungarian Brain Research Progra	Pécs, Hungary	CENTER-TBI Participant + Investigator		
Olli	Tenovuo			Division of Clinical Neurosciences	Turku, Finland	CENTER-TBI Participant + Investigator		
Otesile	Olubukola			Centre for Urgent and Emergency	Sheffield, UK	CENTER-TBI Participant + Investigator		
Paul M.	Parizel			Department of Radiology, Univers	Edegem, Belgium	CENTER-TBI Participant + Investigator		
Peter	Smielewski			Brain Physics Lab, Division of Ne	Cambridge, UK	CENTER-TBI Participant + Investigator		
Peter J.	Hutchinson			Division of Neurosurgery, Departr	Cambridge, UK	CENTER-TBI Participant + Investigator		
Ranjit D.	Singh			Dept. of Neurosurgery, Leiden Ur	The Hague, The Netherl	CENTER-TBI Participant + Investigator		
Renan	Sanchez-Porras			Klinik für Neurochirurgie, Klinikum	Ludwigsburg, Germany	CENTER-TBI Participant + Investigator		
Rico Frederik	Schou			Department of Neuroanesthesia a	Odense, Denmark	CENTER-TBI Participant + Investigator		
Riikka	Takala			Perioperative Services, Intensive	Turku, Finland	CENTER-TBI Participant + Investigator		
Roel P. J.	van Wijk			Dept. of Neurosurgery, Leiden Ur	The Hague, The Netherl	CENTER-TBI Participant + Investigator		
Sophie	Richter			Division of Anaesthesia, Universit	Cambridge, UK	CENTER-TBI Participant + Investigator		
Stefan	Wolf			Department of Neurosurgery, Cha	Berlin, Germany	CENTER-TBI Participant + Investigator		
Steven	Laureys			Cyclotron Research Center , Univ	Liège, Belgium	CENTER-TBI Participant + Investigator		
Thijs	Vande Vyvere			Radiology Department, Antwerp U	Edegem, Belgium	CENTER-TBI Participant + Investigator		
Thomas A.	van Essen			Dept. of Neurosurgery, Leiden Ur	The Hague, The Netherl	CENTER-TBI Participant + Investigator		

*First name, last name, and suffix (if applicable) are required and will appear in PubMed.

***First Name and Middle Initial(s)**	***Last Name**	***Suffix (eg, Jr, III)**	Academic Degrees	Institution	Location (city, state/province, country)	Role or Contribution, eg, chair, principal investigator	Group (if more than 1 Group listed in the byline) and/or Subgroup (eg, Steering Committee)	
Tomas	Menovsky			1. Department of Neurosurgery, Antwerp University Hospital; 2. Department of Translational Neuroscience, Faculty of Medicine and Health Science, University of Antwerp	1. Edegem, Belgium; 2. Antwerp, Belgium	1. CENTER-TBI Participant + Investigator; 2. CENTER-TBI Participant + Investigator		
Tommaso	Zoerle			Neuro ICU, Fondazione IRCCS C	Milan, Italy	CENTER-TBI Participant + Investigator		
Véronique	De Keyser			Department of Neurosurgery, Ant	Edegem, Belgium	CENTER-TBI Participant + Investigator		
Victor	Volovici			Department of Neurosurgery, Era	Rotterdam, Netherlands	CENTER-TBI Participant + Investigator		
Viktória	Tamás			Department of Neurosurgery, Univ	Pécs, Hungary	CENTER-TBI Participant + Investigator		
Vincent	Degos			Anesthesie-Réanimation, Assistan	Paris, France	CENTER-TBI Participant + Investigator		
Vincent	Perlbarg			Anesthesie-Réanimation, Assistan	Paris, France	CENTER-TBI Participant + Investigator		
Virginia	Newcombe			Division of Anaesthesia, Universit	Cambridge, UK	CENTER-TBI Participant + Investigator		
Oliver	Sakowitz			1. Department of Neurosurgery, University Hospital Heidelberg; 2. Klinik für Neurochirurgie, Klinikum Ludwigsburg	1. Heidelberg, Germany; 2. Ludwigsburg, Germany	1.CENTER-TBI Participant + Investigator; 2. CENTER-TBI Participant + Investigator		

*First Name and Middle Initial(s)	*Last Name	*Suffix (eg, Jr, III)	Academic Degrees	Institution	Location (city, state/province, country)	Role or Contribution, eg, chair, principal investigator	Group (if more than 1 Group listed in the byline) and/or Subgroup (eg, Steering Committee)	
Aarno	Palotie			1. Institute for Molecular Medicine Finland, University of Helsinki; 2. Analytic and Translational Genetics Unit, Department of Medicine; Psychiatric & Neurodevelopmental Genetics Unit, Department of Psychiatry; Department of Neurology, Massachusetts General Hospital; 3. Program in Medical and Population Genetics; The Stanley Center for Psychiatric Research, The Broad Institute of MIT and Harvard	1. Helsinki, Finland; 2. Boston, MA, USA; 3. Cambridge, MA, USA	1. CENTER-TBI Participant; 2. CENTER-TBI Participant; 3. CENTER-TBI Participant		
Olav	Roise			1. Division of Orthopedics, Oslo University Hospital; 2. Institue of Clinical Medicine, Faculty of Medicine, University of Oslo	1. Oslo, Norway; 2. Oslo, Norway	1.CENTER-TBI Participant + Investigator + MC; 2.CENTER-TBI Participant + Investigator + MC		
Andrew I.R.	Maas			1. Department of Neurosurgery, Antwerp University Hospital; 2. Department of Translational Neuroscience, Faculty of Medicine and Health Science, University of Antwerp	1. Edegem, Belgium; 2. Antwerp, Belgium	1. CENTER-TBI Participant + Investigator; 2. CENTER-TBI Participant + Investigator		
Herbert	Schoechl			Department of Anaesthesiology a	Salzburg, Austria	CENTER-TBI Associated Participant		
Manuel	Cabeleira			Brain Physics Lab, Division of Ne	Cambridge, UK	CENTER-TBI Participant + Investigator		
Monika	Bullinger			Department of Medical Psycholog	Hamburg, Germany	CENTER-TBI Associated Participant		
Russell L.	Gruen			College of Health and Medicine, A	Canberra, Australia	CENTER-TBI Associated Participant		
Deepak	Gupta			Department of Neurosurgery, Neu	New Delhi-110029, India	CENTER-TBI Associated participant + Investigator		

*First name, last name, and suffix (if applicable) are required and will appear in PubMed.

*First Name and Middle Initial(s)	*Last Name	*Suffix (eg, Jr, III)	Academic Degrees	Institution	Location (city, state/province, country)	Role or Contribution, eg, chair, principal investigator	Group (if more than 1 Group listed in the byline) and/or Subgroup (eg, Steering Committee)	
Rolf	Rossaint			Department of Anaesthesiology, U	Aachen, Germany	CENTER-TBI Associated participant + Investigator		
Agate	Ziverte			Neurosurgery clinic, Pauls Stradir	Riga, Latvia	CENTER-TBI Investigator		
Alex	Manara			Intensive Care Unit, Southmead H	Bristol, Bristol, UK	CENTER-TBI Investigator		
Inigo	Pomposo			Department of Neurosurgery, Hos	Bilbao, Spain	CENTER-TBI Investigator		
Maurizio	Berardino			Department of Anesthesia & ICU,	Torino, Italy	CENTER-TBI Investigator		
Daniel	Rueckert			Department of Computing, Imp	London, UK	CENTER-TBI Participant		
Julia	Mattern			Department of Neurosurgery, Univ	Heidelberg, Germany	CENTER-TBI Participant		
Silke	Schmidt			Department Health and Preventio	Greifswald, Germany	CENTER-TBI Participant		
Valerie	Legrand			VP Global Project Management C	Paris, France	CENTER-TBI Participant		
Visakh	Muraleedharan			Karolinska Institutet, INCF Interna	Stockholm, Sweden	CENTER-TBI Participant + DCTF		
Audny	Anke			Department of Physical Medicine	Tromso, Norway	CENTER-TBI Participant + Investigator		
Cecilia	Åkerlund			Department of Physiology and Ph	Stockholm, Sweden	CENTER-TBI Participant + Investigator		
Krisztina	Amrein			János Szentágothai Research Ce	Pécs, Hungary	CENTER-TBI Participant + Investigator		
Lasse	Andreassen			Department of Neurosurgery, Univ	Tromso, Norway	CENTER-TBI Participant + Investigator		
Nada	Andelic			Division of Clinical Neuroscience,	Oslo, Norway	CENTER-TBI Participant + Investigator		
David	Menon			Division of Anaesthesia, Universit	Cambridge, UK	CENTER-TBI Participant + Investigator + MC		
Nino	Stocchetti			Department of Pathophysiology a	Milano, Italy	CENTER-TBI Participant + Investigator + MC		
Wilco	Peul			Dept. of Neurosurgery, Leiden Ur	The Hague, Netherlands	CENTER-TBI Participant + Investigator + MC		
Fiona	Lecky			1. Centre for Urgent and Emergency Care Research (CURE), Health Services Research Section, School of Health and Related Research (ScHARR), University of Sheffield;<br>2. Emergency Department, Salford Royal Hospital	1. Sheffield, UK;<br>2. Salford, UK	1.CENTER-TBI Participant + Investigator + MC;<br>2.CENTER-TBI Participant + Investigator + MC		

Supplemental Online Content: Nonauthor Collaborators
*First name, last name, and suffix (if applicable) are required and will appear in PubMed.

***First Name and Middle Initial(s)**	***Last Name**	***Suffix (eg, Jr, III)**	Academic Degrees	Institution	Location (city, state/province, country)	Role or Contribution, eg, chair, principal investigator	Group (if more than 1 Group listed in the byline) and/or Subgroup (eg, Steering Committee)	
Giuseppe	Citerio			1. School of Medicine and Surgery, Università Milano Bicocca; 2. NeuroIntensive Care Unit, Department Neuroscience, IRCCS Fondazione San Gerardo dei Tintori	1. Milano, Italy; 2. Milano, Italy	1.CENTER-TBI Participant + Investigator + MC; 2.CENTER-TBI Participant + Investigator + MC		
Ewout W.	Steyerberg			1. Department of Public Health, Erasmus Medical Center-University Medical Center; 2. Dept of Department of Biomedical Data Sciences, Leiden University Medical Center	1. Rotterdam, Netherlands; 2. Leiden, Netherlands	1. CENTER-TBI Participant + MC; 2. CENTER-TBI Participant + MC		
Hester	Lingsma			Department of Public Health, Eras	Rotterdam, Netherlands	CENTER-TBI Participant + MC		
Dashiell	Gantner			ANZIC Research Centre, Monash	Melbourne, Victoria, Aus	Oz ENTER		
Lynnette	Murray			ANZIC Research Centre, Monash	Melbourne, Victoria, Aus	Oz ENTER		
Shirley	Vallance			ANZIC Research Centre, Monash	Melbourne, Victoria, Aus	Oz ENTER		
Tony	Trapani			ANZIC Research Centre, Monash	Melbourne, Victoria, Aus	Oz ENTER		
Anne	Vik			1.Department of Neuromedicine and Movement Science, Norwegian University of Science and Technology, NTNU; 2.Department of Neurosurgery, St.Olavs Hospital, Trondheim University Hospital	1. Trondheim, Norway; 2. Trondheim, Norway	1.CENTER-TBI Investigator; 2.CENTER-TBI Investigator		
Stefan	Winzeck			Division of Anaesthesia, Universit	Cambridge, UK	CENTER-TBI Participant		
