# Supplementary material for: Early vs Late Fixation of Extremity Fractures Among Adults With Traumatic Brain Injury
Source: JAMA Netw Open. 2024 Mar 8;7(3):e241556. doi: 10.1001/jamanetworkopen.2024.1556 (PMC10924246; doi:10.1001/jamanetworkopen.2024.1556)
Supplement: Supplement 3. — Data Sharing Statement [file jamanetwopen-e241556-s003.pdf]

Data Sharing Statement

Zheng. Early vs Late Fixation of Extremity Fractures Among Adults With Traumatic Brain Injury. *JAMA Netw Open*. Published March 08, 2024. doi:10.1001/jamanetworkopen.2024.1556

Data
Data available: No

Additional Information
Explanation for why data not available: The data analyzed in this study was obtained from the CENTER-TBI database; the following licenses/restrictions apply: Upon a reasonable request, access to the dataset must first be reviewed and approved by the CENTER-TBI Management Committee and should be directed to https://www.center-tbi.eu/Data.
